# Amylin Receptor 1 Mutagenesis Revealed a Potential Role of Calcitonin Serine 29 in Receptor Interaction

**DOI:** 10.3390/biomedicines13071787

**Published:** 2025-07-21

**Authors:** Hyeseon Song, Jaehyeok Jang, Minjae Park, Junsu Yun, Jeongwoo Jin, Sangmin Lee

**Affiliations:** Department of Medicinal Biotechnology, College of Health Science, Dong-A University, Busan 49315, Republic of Korea; hyeseon56@naver.com (H.S.); jjh3027006@naver.com (J.J.); sniper4994@naver.com (M.P.); sk8erjunsu@naver.com (J.Y.); jw1603@naver.com (J.J.)

**Keywords:** peptide hormones, calcitonin, amylin, mutagenesis, receptor–ligand binding

## Abstract

**Background:** The amylin receptor is a receptor for the peptide hormone amylin, and its activation is known to reduce body weight. The amylin receptor functions as a heterodimer complex that consists of the calcitonin receptor for peptide hormone calcitonin and an accessary protein. Although the structural information of amylin receptors is currently available, receptor–ligand binding studies that support the peptide binding mode for amylin receptors remain incomplete. **Methods:** Here, we introduced mutagenesis to the amylin receptor 1 extracellular domain and examined mutational effects on peptide binding affinity. We focused on several residues mainly from the peptide-binding pocket (D97, D101, E123, N124, and N135 of the calcitonin receptor). Two well-known peptide ligands for amylin receptors were used for this study: a salmon calcitonin fragment and an antagonist amylin analog AC413 fragment with Y25P mutation. **Results:** Among the introduced mutations, D101A and N135A mutations abolished peptide ligand binding, suggesting that these residues are critical for peptide interaction. The N124A mutation also significantly decreased the peptide binding affinity by more than 8-fold. Intriguingly, the N124D mutation restored the decreased affinity of the salmon calcitonin fragment, while it failed to restore the decreased affinity of the AC413 fragment. Structural analyses suggested that there was a potential role of salmon calcitonin serine 29 in the interaction with aspartate of the N124D mutation. **Conclusions:** This study validates the critical residues of the amylin receptor 1 extracellular domain for the interaction with C-terminal fragments of peptide ligands. This study also suggests that modulating receptor–ligand interaction is feasible by the modification of receptor amino acids near an interacting peptide ligand.

## 1. Introduction

Amylin is a peptide hormone secreted from pancreatic β-cells together with insulin after a meal. It consists of 37 amino acids, and its receptor activation provides benefits for the control of blood glucose and body weight [1]. Amylin receptor activation is known for reducing food appetite, slowing gastric emptying, and reducing glucagon secretion from pancreatic α-cells [1]. Pramlintide, which is a mutated human amylin analog, is clinically available for Type I and II diabetes treatment as adjunct therapy with insulin [2]. Currently, pharmaceutical companies are trying to develop amylin analogs for obesity treatment. Among others, a lipidated amylin and calcitonin receptor dual agonist, cagrilintide, appears to be the most advanced [3] and has been tested in clinical trials for body weight reduction [4,5,6].

The amylin receptor is known as the heterodimer complex of the calcitonin receptor and an accessory protein called receptor activity-modifying protein (RAMP). Recent structural studies clearly showed the heterodimer complex and suggested the binding mode of peptide ligands [7]. The calcitonin receptor is the receptor for another peptide hormone, calcitonin, which consists of 32 amino acids [8]. The calcitonin receptor alone can function as a receptor for the calcitonin to control calcium homeostasis [9]. When the calcitonin receptor forms a complex with RAMP, the complex becomes the amylin receptor. There are three types of RAMP in humans (RAMP1–3). The RAMP1, RAMP2, or RAMP3 complex with the calcitonin receptor functions as amylin receptors 1, 2, or 3, respectively [1]. The amylin receptors 1 and 3 appear to be the main types of amylin receptors since they are known to show selectivity for amylin over calcitonin, compared to amylin receptor 2 [10,11].

Due to the inherent nature of the calcitonin receptor as the main component of the functional amylin receptor, a calcitonin peptide is used as an agonist for amylin receptors. Interestingly, calcitonin originating from salmon is known to bind both human calcitonin and amylin receptors with strong receptor activation potency [12,13]. Accordingly, salmon calcitonin is well-characterized as a dual agonist for calcitonin and amylin receptors and is commonly used for research targeting amylin receptors. Unfortunately, human amylin is reported to form aggregates, and its aggregation can be a potential issue for drug development [14,15]. In addition, the aggregates are known to harm pancreatic β-cells [16]. However, rodent (rat/mouse) amylin is known to have less propensity for forming aggregates than human amylin [17]. Thus, rat amylin is also used in research targeting amylin receptors more frequently than human amylin.

Although the structural studies of human amylin receptors show the binding mode of salmon calcitonin and rat amylin for all three types of amylin receptors, the biochemical and pharmacological studies supporting the molecular interaction between peptide ligands and the receptors are still incomplete. The research question to address in this study is whether introducing mutagenesis to the amylin receptor would help validate the receptor–ligand interaction shown in the recent cryo-electron microscopy structures of amylin receptors. We investigated the mutational effects of the receptor residues located in a peptide-binding pocket on peptide ligand affinity. This mutagenesis study can provide additional lines of evidence for the molecular interaction between peptide ligands and amylin receptors.

This study chose the extracellular domain of amylin receptor 1 and introduced mutagenesis to the residues potentially responsible for peptide ligand binding. We focused on the polar and negatively charged residues of the calcitonin receptor extracellular domain. We used a salmon calcitonin fragment and an antagonist amylin analog AC413 fragment (with Y25P mutation) to investigate their molecular interaction with the mutated receptors. Our results provide insight into the potential role of the peptide residues in the molecular interaction with amylin receptor 1.

## 2. Materials and Methods

### 2.1. Reagents

Dulbecco’s Modified Eagle Medium (DMEM) including 4.5 g/L glucose, L-glutamine, and sodium pyruvate was purchased from Cytiva (Cat.# SH30243.01, South Logan, UT, USA) to culture human embryonic kidney (HEK) 293 cells. Fetal bovine serum (Gibco™ FBS, Cat.# 16000-044) was purchased from ThermoFisher Scientific (Waltham, MA, USA) for mammalian cell culture. NEBuilder^®^ HiFi DNA Assembly Master Mix and several restriction enzymes required for DNA cloning were purchased from New England Biolabs (Ipswich, MA, USA). Additionally, 4–15% Mini-PROTEAN TGX Stain-Free Precast Gels were purchased from Bio-Rad (Hercules, CA, USA) to confirm the purified receptor proteins. All other reagents were purchased from Sigma-Aldrich (St. Louis, MO, USA), unless otherwise noted.

### 2.2. Cell Line and Bacteria Cells Used

HEK293 cells (KCLB number, 21,573) were purchased from Korean Cell Line Bank (KCLB, Seoul, Republic of Korea) to express wild-type and mutated amylin receptor 1 extracellular domains. This cell line has been authenticated from the KCLB, and cells with passage numbers less than 50 were used for this study. DH5α Chemically Competent *E. coli* cells (Cat.# CP011) used for DNA cloning were purchased from Enzynomics (Daejeon, Republic of Korea).

### 2.3. Expression Plasmids

pHLsec-based vectors were used to express wild-type and mutated amylin receptor 1 extracellular domains as secreted proteins to cell culture media [18]. The plasmid DNA construct of the wild-type amylin receptor 1 extracellular domain, pHLsec/hRAMP1.24–111-(GSA)_3_-hCTR.34–141-H_6_ (H-pSL005) was previously described [19]. The following DNA vector plasmids were made for this study: pHLsec/hRAMP1.24–111-(GSA)_3_-hCTR.34–141.D97A-H_6_ (D-pSL009), pHLsec/hRAMP1.24–111-(GSA)_3_-hCTR.34–141.D97E-H_6_ (D-pJS001), pHLsec/hRAMP1.24–111-(GSA)_3_-hCTR.34–141.D101A-H_6_ (D-pJJ003), pHLsec/hRAMP1.24–111-(GSA)_3_-hCTR.34–141.D101E-H_6_ (D-pJJ002), pHLsec/hRAMP1.24–111-(GSA)_3_-hCTR.34–141.E123A-H_6_ (D-pJJ004), pHLsec/hRAMP1.24–111-(GSA)_3_-hCTR.34–141.E123D-H_6_ (D-pJJ001), pHLsec/hRAMP1.24–111-(GSA)_3_-hCTR.34–141.N124A-H_6_ (D-pJS005), pHLsec/hRAMP1.24–111-(GSA)_3_-hCTR.34–141.N124D-H_6_ (D-pJS006), pHLsec/hRAMP1.24–111-(GSA)_3_-hCTR.34–141.N135A-H_6_ (D-pJW001), and pHLsec/hRAMP1.24–111-(GSA)_3_-hCTR.34–141.N135D-H_6_ (D-pJS002). The mutations were introduced to the calcitonin receptor extracellular domain by using DNA Assembly reaction. Coding sequences of the receptor expression vectors were confirmed with Sanger sequencing by Bionics (Seoul, Republic of Korea). DNA plasmid vectors were extracted and were purified from bacterial cells with NucleoBond® Extra Midi Plus kit (Macherey-Nagel, Germany). The purified DNA vectors were stored at −20 °C until their use for mammalian cell transfection.

### 2.4. Receptor Protein Purification

The overall experimental procedure of reconstituting the functional amylin receptor 1 extracellular domain (ECD) as the fusion protein of RAMP1 ECD and the calcitonin receptor ECD was previously described [19,20]. The wild-type and mutated amylin receptor 1 ECDs were expressed from HEK293 cells with transient transfection with polyethylenimine. The expressed receptor proteins were secreted to cell culture media for 4 days at 37 °C. The media were collected for the receptor protein purification. Initial dialysis was performed with the dialysis buffer (Tris 25 mM (pH 7.4), NaCl 150 mM, and imidazole 10 mM) overnight. The next day, immobilized metal affinity column chromatography (IMAC) trapping a multi histidine-tag attached to the receptor protein was performed by using HisTrap^TM^ HP 5 mL (Cytiva, Cat.#17524801) with IMAC Buffer A (Tris 50 mM (pH 7.4), NaCl 150 mM, glycerol (*v*/*v*) 10%, and imidazole 25 mM) and Buffer B (Tris 50 mM (pH 7.4), NaCl 150 mM, glycerol (*v*/*v*) 10%, and imidazole 500 mM). Then, a size exclusion column chromatography (SEC) was performed with SEC column (Cytiva, HiLoad^®^ 16/600 Superdex^®^ 200 pg) and SEC buffer (Tris 50 mM (pH 7.4), NaCl 150 mM, and glycerol (*v*/*v*) 10%) as previously described [19]. As the final step, the purified receptor proteins were dialyzed to storage buffer (HEPES 25 mM (pH 7.4), NaCl 150 mM, and glycerol (*v*/*v*) 50 %) and were stored at −60 °C until their use for the receptor–ligand binding assay. All purification procedures were performed at 4 °C.

### 2.5. Synthetic Peptides

**Figure 1:**
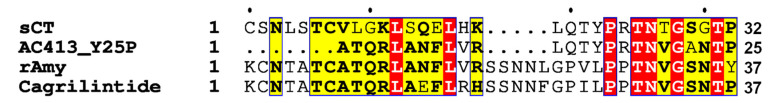
Sequence alignment of peptide probes used in the current study. Rat amylin (rAmy) and a promising amylin receptor activator cagrilintide were added for the sequence alignment. sCT, salmon calcitonin. AC413 Y25P is an amylin receptor antagonist AC413 with Y25P mutation. Cagrilintide is a lipidated peptide. Lipidation of cagrilintide was hidden for clarity. Sequences were aligned with ClusterX2.1, and ESPript 3.0 was used to make figure representation. Similar residues are shown in black bold characters and in yellow boxes. The same residues for all four peptides are shown in white bold characters and in red boxes.

Two peptide probes labeled with fluorescein isothiocyanate (FITC) were used for this study: FITC-labeled salmon calcitonin (sCT) fragment(22–32) and FITC-labeled AC413(6–25) with Y25P mutation. These peptide probes were custom-synthesized from Genscript (Piscataway, NJ, USA). These peptides were used as the probes for the receptor–ligand binding assay. These peptides were HPLC-purified with at least 85% purity by Genscript (Piscataway, NJ, USA). Mass spectrometry performed at Genscript confirmed the correct molecular mass of the synthesized peptides. The extinction coefficient of FITC (63,000 M^−1^·cm^−1^ at 495 nm, pH 7.0) was used to calculate the concentration of the peptides. The sequence of these peptide probes is shown in Figure 1.

### 2.6. Fluorescence Polarization/Anisotropy (FP) Receptor–Ligand Binding Assay

The overall procedure of the FP binding assay with a saturation binding format was previously described [21]. A total of 10 nM FITC-labeled sCT(22–32) and 10 nM FITC-labeled AC413(6–25) with Y25P mutation were used for the receptor–ligand binding assay. For the saturation binding format, the non-linear regression curves were generated by using user-defined equations that were previously described for affinity calculation [21,22]. FITC-labeled peptide probes were incubated for 1 h at room temperature with the purified receptor protein prior to fluorescence signal measurement. A BioTek Synergy H1 microplate reader (Agilent, Santa Clara, CA, USA) was used to evaluate fluorescence polarization/anisotropy. Background signal from the plates was subtracted for accurate calculation. G factor (0.95) was used for the signal from FITC-labeled sCT(22–32) to set the polarization (mP) of the free FITC-peptide probes close to 50 mP. For the AC413(6–25) Y25P probe, G factor (1) was used. When total fluorescence intensity of the FITC-labeled probes was altered with receptor binding by more than 10% from the starting fluorescence value, the anisotropy was adjusted to reflect the fluorescence change as previously described [21]. PRISM 5.0 (Version 5.0, GraphPad software, San Diego, CA, USA) was used for analyzing receptor–ligand binding for this study, and this relatively old version was still able to predict non-linear regression curves for the FP assay as previously reported [19,21]. PRISM 5.0 was fully functional, with the user-defined equations used predicting K_D_ values from fluorescence polarization/anisotropy. Nevertheless, a newer version of PRISM is available, and using a recent version of PRISM would be desirable for potentially more accurate prediction.

For the representative receptor–ligand binding curves in the figures, the mean anisotropy of two technical replicates at each receptor concentration is shown, and the S.E.M. of the anisotropy of the two replicates is presented as error bars. When the error bars were shorter than the height of symbols, they were hidden in the representative curves. At least three independent experiments were performed to obtain at least three pK_D_ (-log_10_K_D_) values of the peptide probes. Mean and standard deviation (S.D.) of the pK_D_ values were calculated and are shown in this study.

### 2.7. Homology Model Structures

The crystal structure of calcitonin receptor ECD with a salmon calcitonin fragment (PDB 6PFO) [23] and the cryo-EM structure of amylin receptor 1 with rat amylin (PDB 7TYF) [7] were used for this study. SWISS-MODEL (available in Expasy web server) was used for making the hypothetical models of the mutated amylin receptor 1 structures [24]. Amino acid sequences of the full-length human calcitonin receptor (P30988, UniProt.org) including one or two mutations and human RAMP1 (O60894, UniProt.org) were used for building mutated amylin receptor 1 structures. SWISS-MODEL found that the cryo-EM structure of amylin receptor 1 bound with cagrilinitide (PDB 9BP3) [25] was the most suitable structure for the model building for all instances of mutated amylin receptor 1. For the peptide ligands, at least 30 amino acids were needed for model building with SWISS-MODEL. Accordingly, rat amylin(1–26)-salmon calcitonin(22–32) fusion peptide and rat amylin(1–7)-AC413(1–25) Y25P fusion peptide were used for peptide model building bound for the mutated amylin receptor 1. These models were visualized with PyMOL (The PyMOL Molecular Graphics System, Version 2.3.0 Schrödinger, LLC., New York, NY, USA). The distance between amino acid residues in proximity was measured with the measurement option available in PyMOL.

### 2.8. Statistical Analysis

One-way ANOVA with Dunnett’s post hoc test (the wild-type receptor was used as a control group) or with Tukey’s post hoc test was used for statistical analysis. A relatively old version of PRISM (Version 5.0, GraphPad software, San Diego, CA, USA) was used, but it was fully capable of addressing statistical analyses for this study. The results with *p* < 0.05 were considered a statistically significant difference.

## 3. Results

### 3.1. Rationale of Selecting Amylin Receptor Residues for Mutagenesis

It has been reported that the accessory protein RAMP has minimal interaction with peptide ligands at amylin receptors [7]. Most of the peptide-binding regions (or of the peptide-binding pocket) were provided by the calcitonin receptor. The C-terminal region of peptide agonists consisting of approximately 11 amino acids was responsible for direct binding of the calcitonin receptor ECD [19,23]. Thus, we decided to introduce mutations to the calcitonin receptor ECD to investigate the molecular interaction between the C-terminal fragment of peptide ligands and amylin receptor 1 ECD.

**Figure 2:**
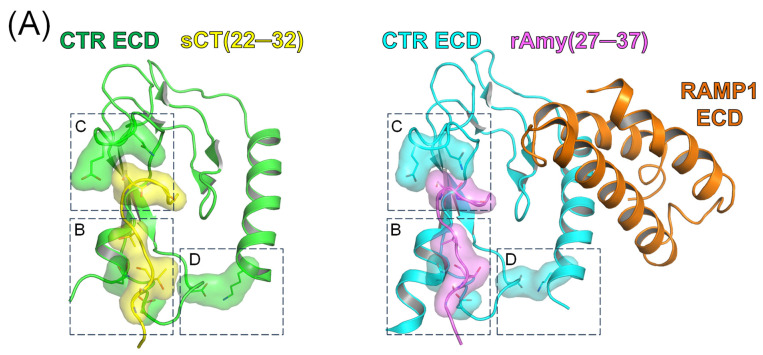

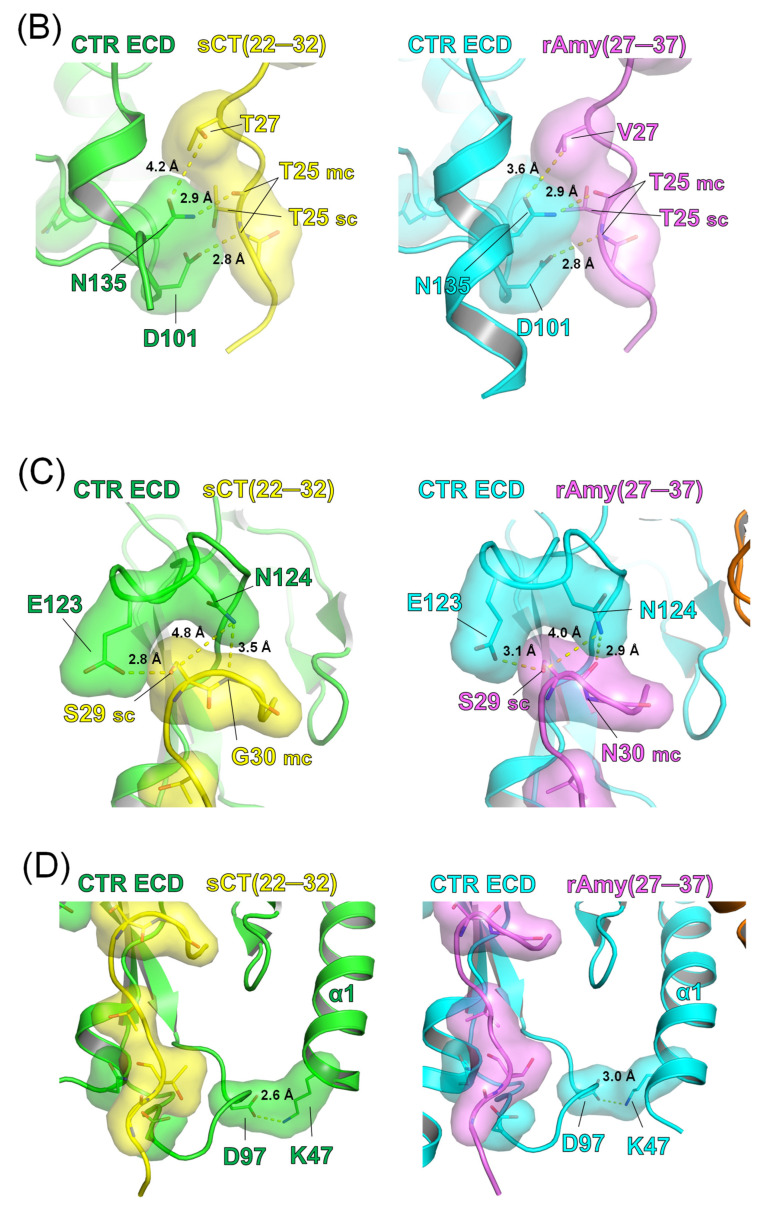
Structures of calcitonin and amylin receptor ECDs. (**A**) A crystal structure of human calcitonin receptor ECD with salmon calcitonin fragment (PDB 6PFO) (left) and a cryo-electron microscopy structure of human amylin receptor 1 ECD with rat amylin (PDB 7TYF) (right). Boxes with dotted lines indicate the areas for blown-up views in the following panels. The letter of the upper left side of the dotted box indicates the following blown-up panel. (**B**–**D**) Blown up views of the boxes with dotted lines. For panel B, the structures were rotated around the Y axis by 100 degrees for the clear view of receptor–ligand interaction. The receptor residues for mutagenesis and the potentially interacting residues of peptide ligands are shown in stick and surface representations. The distance was measured by using a measurement option available in PyMOL. mc, main chain. sc, side chain. α1, N-terminal α1 helix of the calcitonin receptor ECD. sCT, salmon calcitonin. rAmy, rat amylin. CTR, calcitonin receptor. RAMP1, receptor activity-modifying protein 1. ECD, extracellular domain.

Previous studies with alanine-scanning mutagenesis of C-terminal calcitonin and amylin peptide fragments suggested the critical residues of peptide ligands for receptor interaction [20,26]. Salmon calcitonin threonine 25 and threonine 27 (corresponding to rat amylin threonine 30 and valine 32 when aligned) appeared essential for receptor interaction since the alanine mutation introduced to those peptide residues abolished the binding for calcitonin and amylin receptor ECDs [20,26]. We chose calcitonin receptor aspartate 101 (D101) and asparagine 135 (N135) residues as potential interaction sites for the above peptide residues (Figure 2A,B). Interestingly, calcitonin serine 29 residue (corresponding to rat amylin serine 34 when aligned) appeared dispensable for receptor ECD binding [20,26]. Alanine-scanning mutagenesis showed that alanine mutation of salmon calcitonin serine 29 increased binding for the receptor ECDs [20]. The competition binding assay also reported that rat amylin serine 34 to alanine mutation significantly increased the peptide affinity for amylin receptor 1 ECD by 3-fold [26]. To confirm the potential role of calcitonin serine 29 (rat amylin serine 34) in receptor interaction, the calcitonin receptor glutamine 123 (E123) and asparagine 124 (N124) residues were selected for mutagenesis as the potential interaction sites with calcitonin serine 29 (Figure 2C).

Aspartate 97 (D97) of the calcitonin receptor was also selected, and its role in peptide ligand binding was examined. Although this D97 residue did not directly participate in the peptide ligand binding (Figure 2D), D97 was shown to interact with the N-terminal α1 helical region of the calcitonin receptor ECD. Lysine 47 located in the α1 helical region of the calcitonin receptor appeared to form a salt bridge interaction with D97 (Figure 2D) [7,23]. This interaction may participate in establishing proper conformation of calcitonin receptor ECD, and the potential effects of disrupting this interaction on peptide binding were examined by mutating the D97 residue.

We used two peptide probes: a salmon calcitonin (sCT) (22–32) fragment and an amylin receptor antagonist AC413(6–25) fragment with Y25P mutation. These two probes were FITC-labeled for the fluorescence polarization peptide-binding assay. The sequence alignment including these two probes is shown in Figure 1. sCT(22–32) and eleven C-terminal amino acids of AC413(6–25) Y25P shared most of the amino acids. Interestingly, some of the peptide amino acids responsible for binding receptor ECDs were different between sCT(22–32) and AC413(6–25) Y25P (Figure 1). sCT(22–32) had T27, S29, and G30, while the corresponding residues of AC413(6–25) Y25P were V20, A22, and N23. Other C-terminal residues responsible for direct receptor ECD binding were conserved in sCT(22–32) and AC413(6–25) Y25P.

### 3.2. Mutational Effects of Calcitonin Receptor D101 and N135 Residues on Peptide Probe Affinity

**Figure 3:**
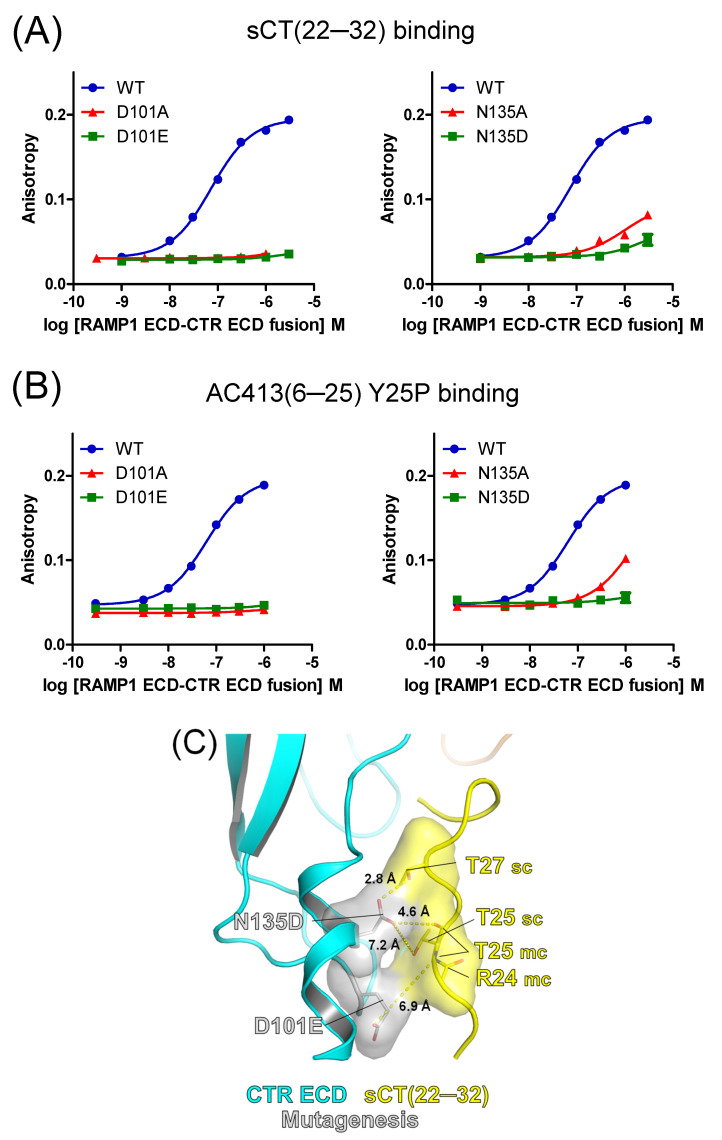
Peptide probe binding for the amylin receptor 1 ECD with the mutations at D101 and N135 residues. (**A**) FITC-sCT(22–32) binding for the amylin receptor 1 ECD with calcitonin receptor D101A (left), D101E (left), N135A (right), and N135D (right) mutations. (**B**) FITC-AC413(6–25) Y25P binding curves with the amylin receptor 1 ECD with calcitonin receptor D101A (left), D101E (left), N135A (right), and N135D (right) mutations. Representative binding curves from at least three independent experiments were shown. (**C**) A hypothetical model of sCT(22–32) and the amylin receptor 1 ECD with calcitonin receptor D101E and N135D mutations. Calcitonin receptor D101E and N135D mutated residues and potentially interacting sCT(22–32) R24, T25, and T27 residues were shown as stick and surface representation. CTR, calcitonin receptor. sCT, salmon calcitonin. sc, side chain. mc, main chain.

As expected, D101A and N135A mutations of the calcitonin receptor markedly decreased the binding affinity of sCT(22–32) and AC413(6–25) Y25P (Figure 3A,B and Table 1). These results suggest that D101 and N135 residues were integral to the interaction with the peptide ligands. Chemical interaction of these residues with peptide ligands may be critical for the peptide binding affinity such that mutations of these two residues dramatically decreased the affinity of the peptide ligands. These mutations also hold a possibility of producing misfolded receptor ECD proteins. Nevertheless, size exclusion chromatography elution profiles of the mutated ECDs (Figure S1) and their SDS-PAGE results (Figure S2) were quite comparable to those of the wild-type receptor. In addition, peak elution volumes of wild-type and mutated receptor proteins from size exclusion chromatography were also similar (Table S1). These results suggest that the mutated receptor ECD proteins are likely to have similar conformation to the wild-type receptor.

The D101E mutation failed to recover the binding affinity of sCT(22–32) (Figure 3A and Table 1). Glutamate of the D101E mutation retained the negative charge, while glutamate had one more methylene group than aspartate (Figure 3C). A bit longer side chain of glutamate appeared to be located away from the sCT(22–32) T25 main chain (Figure 3C). Aspartate of the N135D mutation retained the same length of the side chain as asparagine, while a negative charge was placed at the end. Although the N135D mutation appeared to be in the proximity of the T27 side chain and the T25 main chain within 3~5 Å distance (Figure 3C), the mutation failed to recover the binding affinity of sCT(22–32) and AC413(6–25) Y25P. These results suggest that the polar residue of N135 is quite optimal for the peptide ligand interaction and that the negatively charged aspartate residue may create repulsion against the tested peptide ligands. In addition, it cannot be ruled out that the N135D mutation disrupts the conformation of the receptor ECD such that the peptide-binding pocket was altered and was unable to interact with the peptide probes.

**Figure 4:**
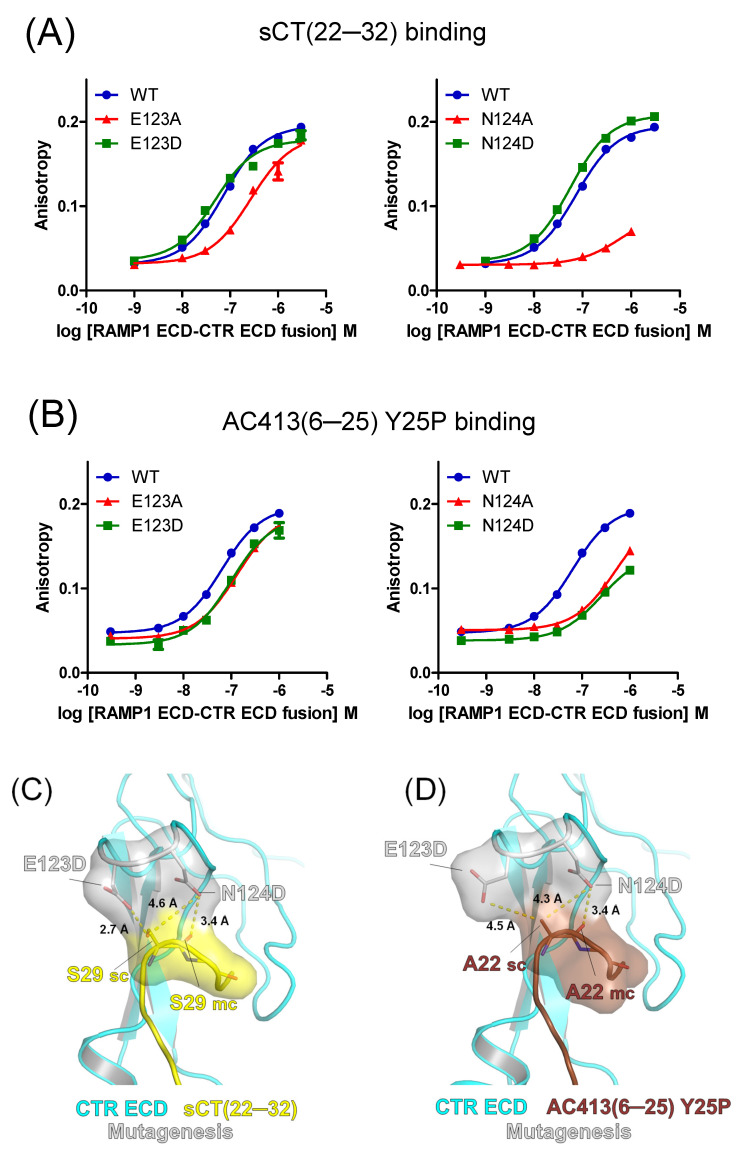
Peptide probe binding for the amylin receptor 1 ECD with the mutations at E123 and N124 residues. (**A**) FITC-sCT(22–32) binding for the amylin receptor 1 ECD with calcitonin receptor E123A (left), E123D (left), N124A (right), and N124D (right) mutations. (**B**) FITC-AC413(6–25) Y25P binding curves with the amylin receptor 1 ECD with calcitonin receptor E123A (left), E123D (left), N124A (right), and N124D (right) mutations. Representative binding curves from three independent experiments are shown. Peptide-binding curves for the wild-type amylin receptor 1 ECD used for Figure 3 are shown again. (**C**,**D**) Hypothetical models of the amylin receptor 1 ECD with calcitonin receptor E123D and N124D mutations. Calcitonin receptor E123D and N124D mutated residues and potentially interacting sCT(22–32) S29 and AC413(6–25) Y25P A22 residues are shown as stick and surface representations. sCT(22–32) G30 and the main chain of AC413(6–25) Y25P N23 are also shown as stick and surface representations. CTR, calcitonin receptor. sCT, salmon calcitonin. sc, side chain. mc, main chain.

**Figure 5:**
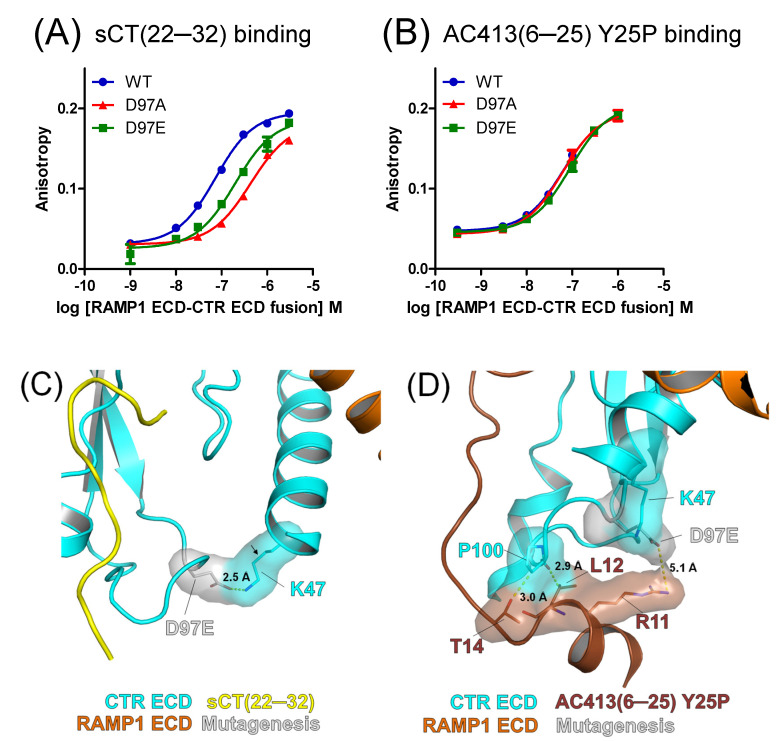
Peptide probe binding for the amylin receptor 1 ECD with the mutations at the D97 residue. (**A**) FITC-sCT(22–32) binding for the amylin receptor 1 ECD with calcitonin receptor D97A and D97E mutations. (**B**) FITC-AC413(6–25) Y25P binding curves with the amylin receptor 1 ECD with calcitonin receptor D97A and D97E mutations. Representative binding curves from three independent experiments are shown. Peptide binding curves for the wild-type amylin receptor 1 ECD used for Figure 3 are shown again. (**C**,**D**) Hypothetical models of peptide probes and the amylin receptor 1 ECD with the calcitonin receptor D97E mutation. Calcitonin receptor D97E, K47, and P100 and nearby AC413(6–25) Y25P peptide R11, L12, and T14 residues are shown as stick and surface representations. For (**C**), an arrow indicates a less common *cis*-conformation of the calcitonin receptor K47 side chain. CTR, calcitonin receptor. sCT, salmon calcitonin. RAMP1, receptor activity-modifying protein 1.

Caution should be used for interpreting the distances between amino acids shown in the hypothetical models including those in the following figures (Figure 4C,D and Figure 5C,D) since the structures of the mutated residues were not from experimental determination but were from computational modeling.

### 3.3. Mutational Effects of Calcitonin Receptor E123 and N124 Residues on Peptide Probe Affinity

The E123A mutation significantly decreased the affinity of sCT(22–32) by 4.5-fold (Figure 4A and Table 1). The N124A mutation also markedly decreased the sCT(22–32) affinity by more than 10-fold, suggesting that the N124 residue is critical for sCT(22–32) binding (Figure 4A). The binding affinity of AC413(6–25) Y25P was moderately decreased by the E123A mutation by 2.3-fold. The N124A mutation markedly decreased the AC413(6–25) Y25P affinity by 8.2-fold. These results were comparable to the mutational effects on sCT(22–32) binding affinity, confirming the critical role of the N124 residue for peptide ligand binding. Based on the previous report that the side chain of calcitonin S29 was dispensable for receptor ECD interaction [20], E123 and N124 residues of the calcitonin receptor appeared to interact with the main chain (backbone) of calcitonin (Figure 2C).

Interestingly, the E123D and N124D mutations fully restored the decreased binding affinity of sCT(22–32) (Figure 4A and Table 1). In contrast to the results with sCT(22–32), the E123D and N124D mutations failed to restore the decreased binding affinity of AC413(6–25) Y25P (Figure 4B and Table 1). It appeared that serine 29 of sCT(22–32) was in the proximity of aspartate residues of E123D and N124D mutations within 3~5 Å distance in the hypothetical model (Figure 4C). Serine 29 of sCT(22–32) may interact with the aspartate residues of the E123D and N124D mutations (Figure 4C). In contrast, AC413(6–25) Y25P has alanine 22 as the corresponding residue to the serine 29 of sCT(22–32). Although alanine 22 of AC413(6–25) Y25P was still located within 3~5 Å distance from the aspartate residues of E123D and N124D mutations, the alanine would not form any polar interaction with the mutated residues (Figure 4D). The presence or absence of the hydroxy group between serine 29 of sCT(22–32) and alanine 22 of AC413(6–25) Y25P may explain the different binding results shown in Figure 4A,B. Nevertheless, caution should be used to suggest a binding mechanism based on these hypothetical models.

### 3.4. Mutational Effects of the Calcitonin Receptor D97 Residue on Peptide Probe Affinity

The calcitonin receptor D97 residue was located away from the peptide-binding pocket (Figure 2D). Apparently, it would not provide direct contact to peptide ligands (Figure 2D). The negatively charged D97 residue appeared to interact with a positively charged K47 of the N-terminal α1 helix of the calcitonin receptor through salt interaction (Figure 2D). We hypothesized that chemical interaction between D97 and α1 helix K47 contributes to the conformation of the calcitonin receptor ECD and that its interruption would have a negative effect on peptide ligand binding. As expected, the D97A mutation significantly decreased sCT(22–32) binding affinity by 6.8-fold (Figure 5A and Table 1), supporting the indirect role of the D97 residue in sCT(22–32) binding affinity. The D97E mutation partially restored the decreased affinity of sCT(22–32) (Figure 5A and Table 1). Interestingly, the affinity of AC413(6–25) Y25P was not significantly changed either by the D97A or D97E mutation (Figure 5B and Table 1).

The hypothetical model of amylin receptor 1 with the calcitonin receptor D97E mutation suggests that the D97E mutation still can interact with the K47 residue through salt interaction. The glutamate side chain of the D97E mutation is a bit longer than aspartate, while it retains the negative charge at the end. The side chain of the K47 residue appeared to adopt a less common *cis*-conformation to form a salt interaction with glutamate of the D97E mutation (Figure 5C).

A much longer length of AC413(6–25) Y25P (20 amino acids) than sCT(22–32) (11 amino acids) appeared to provide additional sites for calcitonin receptor interaction (Figure 5D). The hypothetical model with AC413(6–25) Y25P showed that L12 and T14 residues were in the proximity of calcitonin receptor P100 within 3 Å distance. This potential interaction between AC413(6–25) Y25P and the calcitonin receptor P100 may weaken the detrimental effect of the D97A mutation on peptide ligand binding. The glutamate of the D97E mutation appeared to be located 5 Å away from the positively charged R11 of AC413(6–25) Y25P, making the glutamate less likely to form a salt interaction with R11 (Figure 5D). Nevertheless, the hypothetical models shown in Figure 5C,D should be viewed with caution since these models lack supporting experimental evidence.

## 4. Discussion

### 4.1. Effects of FITC Labeling to the Calcitonin Fragment on Receptor ECD Binding

The salmon calcitonin C-terminal fragment was reported to have micromolar affinity (K_I_ 1~3 μM) both for calcitonin and amylin receptor ECDs [19]. The fluorescein isothiocyanate (FITC)-labeled salmon calcitonin fragment (FITC-sCT(22–32)) was used as a peptide probe for the receptor–ligand binding assay in the current study. FITC addition to the N-terminus of an amylin receptor antagonistic peptide AC413(6–25) Y25P was previously shown to increase the peptide affinity for receptor ECD binding by more than 10-fold [21]. Based on the binding affinity of FITC-sCT(22–32) measured in the current study, FITC labeling also appeared to increase the binding affinity of sCT(22–32) for the amylin receptor 1 ECD. The mean pK_D_ of FITC-sCT(22–33) for the wild-type amylin receptor 1 ECD was 7.09 (K_D_ 81 nM, Table 1). Compared to the previously reported K_I_ value of non-labeled sCT(22–32) for the amylin receptor 1 ECD (K_I_ 2.5 μM) [19], FITC-labeled sCT(22–32) showed more than 30-fold stronger affinity for amylin receptor 1 ECD (K_D_ 81 nM). Although FITC labeling is inevitable for a fluorescence polarization peptide binding assay, FITC labeling to peptide probes may generate additional sites for receptor interaction and can prevent accurate measurement of peptide affinity for receptor proteins.

Supplemental Figure S3 shows the results of the competition binding assay with non-labeled sCT(22–32) and amylin receptor 1 ECDs. As expected, the affinity of non-labeled sCT(22–32) (K_I_ 891 nM) was weaker than the affinity of the FITC-labeled version (K_D_ 81 nM). However, the pattern of the E123A and E123D mutational effects was conserved. These results suggest that FITC labeling may not participate in the peptide ligand interaction happening at the binding pocket of amylin receptor 1 ECD. However, we cannot rule out the possibility that FITC labeling might affect peptide ligand interaction with the mutated receptor ECDs.

### 4.2. Limitation of the Current Study in Interpreting Peptide Ligand Interaction

**Figure 6:**
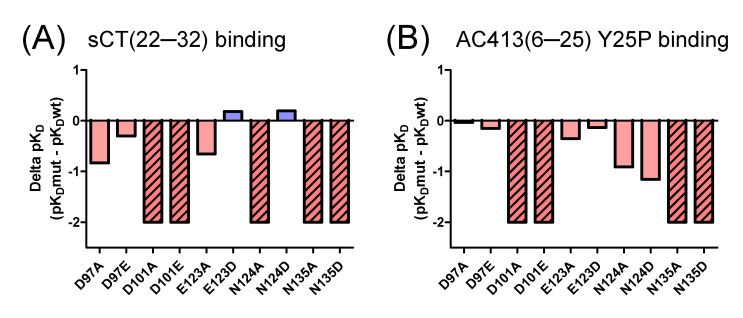
Peptide affinity changes for the amylin receptor 1 ECD by the tested mutations. (**A**,**B**) FITC-sCT(22–32) and FITC-AC413(6–25) Y25P affinity changes, respectively. Delta pK_D_ of peptide probes was calculated by subtracting the mean pK_D_ for the wild-type amylin receptor 1 ECD from the mean pK_D_ for the mutated amylin receptor 1 ECD. When the receptor mutation markedly decreased the peptide binding by more than 100-fold such that the pK_D_ of peptide probes was unable to be determined (shown as N.D. in Table 1), delta pK_D_ was represented as −2, and the corresponding bar graphs are shown with diagonal lines. Delta pK_D_ with negative values is shown in red, while delta pK_D_ with positive values is shown in light blue.

Figure 6 compares the affinity changes in two peptide probes by receptor mutagenesis. Mutating calcitonin receptor D101 and N135 residues dramatically decreased the affinity of both peptide probes, indicating that the participation of D101 and N135 in the peptide interaction was critical. Alanine mutations of E123 and N124 significantly decreased the peptide affinity by more than 2-fold. Interestingly, the decreased affinity of sCT(22–32) was restored by E123D and N124D mutations, while AC413(6–25) Y25P affinity was not restored by the same mutations (Figure 4A,B). The sequence alignment of these peptide probes suggests that serine 29 of sCT(22–32) and the corresponding alanine 22 of AC413(6–25) Y25P may make the differential effects mediated by the E123D and N124D mutations. Hypothetical models of the mutated amylin receptor 1 ECDs with the peptide probes provide insight into the potential mechanism for the affinity changes (Figure 4C,D).

Nevertheless, careful consideration should be placed in the interpretation of our results due to some limiting factors. Our study used purified receptor ECDs and FITC-labeled peptide probes. The functional amylin receptor 1 ECD was reconstituted by making a fusion protein with RAMP1 ECD and calcitonin receptor ECD [19,20]. These two ECDs were tethered with a linker. These proteins were produced from HEK293 cells where the complex N-glycosylation machinery was available, such that the produced proteins were all N-glycosylated (Figure S3). This tethering strategy produced functional N-glycosylated amylin receptor 1 ECDs. Our study focused on the receptor ECD interaction with the C-terminal fragment of the peptide probes, especially the last 11 amino acids directly responsible for ECD interaction. Thus, interpretation of our results should be limited to receptor ECDs and to peptide C-terminal fragments. Our study does not guarantee the interaction of full-length peptide agonists with the full-length amylin receptor 1 including a receptor transmembrane domain. The potential role of calcitonin S29 in amylin receptor 1 interaction should be interpreted with the amylin receptor 1 ECD and peptide fragments used in this study. Our results were confined to the interaction between purified receptor ECDs and peptide ligand fragments, and their direct application to the full-length receptor and full-length peptide ligands is not suitable. There is one report available with full-length human amylin and the role of each amino acid in amylin receptor activation [27]. However, additional receptor–ligand interaction studies are needed for the validation of the full-length amylin peptide pharmacophore.

Mutagenesis itself may produce abnormal conformation of the receptor ECDs. Although introducing mutagenesis is a common strategy for investigating receptor–ligand interaction, it can potentially generate additional effects on receptor conformation. For several mutations such as D101A, D101E, N135A, N135D, and N124A, we were unable to obtain meaningful peptide binding at the tested concentrations. Consequently, the K_D_ values were not determined due to suboptimal prediction with the available binding data. It is possible to state that the decrease in peptide ligand affinity may result from the mutagenesis by altering overall receptor conformation. In addition, potential instability and aggregation of the mutated receptor ECDs might affect peptide ligand interaction.

Mutagenesis introduced to CTR ECD may also affect the interaction between RAMP1 ECD and CTR ECD. Although RAMP1 ECD was shown to have limited contact to the C-terminal residue of peptide ligands [7], RAMP ECD was reported to allosterically modulate its interacting receptor ECD conformation [28,29]. It is also possible that the allosteric modulation of RAMP1 ECD on CTR ECD conformation may not be functional for the mutated CTR ECD. This potential malfunction may affect peptide ligand binding affinity for the amylin receptor 1 ECD.

### 4.3. Alanine-Scanning Mutagenesis Studies on Peptide Interaction with Amylin Receptor 1

Receptor–ligand interaction studies with alanine-scanning mutagenesis have been reported for amylin receptors. Lee et al. first introduced alanine-scanning mutagenesis to calcitonin and AC413 peptides and examined the role of each amino acid in the interaction with amylin receptor 1 ECD [20]. They reported critical amino acids of peptide ligands for amylin receptor 1 ECD binding. Their results were supported by the following structural studies with the calcitonin receptor ECD and salmon calcitonin fragments [23,30]. Recently, Lee S introduced alanine-scanning mutagenesis to a rat amylin fragment and reported the role of each amino acid in amylin receptor ECD interaction [26]. The binding results helped design multiple mutated analogs of the rat amylin fragment with enhanced affinity (over 100-fold) for the amylin receptor 1 ECD [26]. Bower et al. used human amylin and introduced alanine-scanning mutagenesis to human amylin [27]. They reported the molecular interaction of the mutated human amylin with amylin receptors by measuring amylin receptor activation in a cell-based system. The study found the potential human amylin residues with introduced mutations such as A5S, Q10A, and V17A significantly increased the amylin receptor activation potency.

Several receptor–ligand interaction studies introducing mutagenesis to calcitonin and amylin receptors are also available [8,29,31,32,33]. Among these previous reports, an extensive receptor–ligand interaction study was reported where alanine-scanning mutagenesis was applied to the upper segment of transmembrane helix 1 (TM1) of the calcitonin receptor and its extension into the receptor ECD [31]. Although the study focused on TM1, some residues of calcitonin receptor ECD were mutated and were tested for peptide ligand interaction. When the N135 residue was mutated to alanine, the cell-surface expression level of the calcitonin receptor was not significantly changed compared to the wild-type receptor level. However, the calcitonin receptor N135A mutation abolished the binding of sCT(8–32), human calcitonin, and porcine calcitonin for the calcitonin receptor. These results were consistent with the current study, suggesting that the calcitonin receptor N135 residue was critical for peptide ligand binding. Despite significant decreases in peptide affinity by the N135A mutation, calcitonin receptor activation mediated by calcitonin was able to be measured. The N135A mutation decreased functional affinity (pK_A_) for cAMP production and for the phosphorylation of extracellular signal-regulated kinase (ERK), but by less than 10-fold [31].

Gingell et al. addressed the receptor interaction with human calcitonin and rat amylin by introducing mutations to the peptide-binding pocket of the calcitonin receptor and amylin receptor 1 ECDs [29]. The peptide potency for calcitonin receptor and amylin receptor 1 activation was investigated by measuring cAMP production. Multiple residues located in the peptide-binding pocket were tested for peptide-mediated receptor activation. They reported the mutational effects of D101A and N124A that were used in the current study. Consistent with our binding results, the D101A mutation markedly decreased the potency of human calcitonin by 17-fold for calcitonin receptor activation. And the D101A mutation also decreased the potency of rat amylin by 68-fold for amylin receptor 1 activation. However, rat amylin potency for the calcitonin receptor activation was not significantly altered by the D101A mutation.

Regarding the N124A mutation, our results showed significant decreases in peptide ligand affinity by more than 8-fold. Nevertheless, Gingell et al. reported that the N124A mutation did not significantly change the potency of human calcitonin and rat amylin for their receptor activation [29]. The N124 residue of the calcitonin receptor appeared dispensable for amylin receptor 1 activation mediated by calcitonin or amylin. The affinity decreases for receptor ECD by 8-fold and more may be insufficient to produce the potency decrease in the activation of the full-length calcitonin and amylin receptors. Potentially, this inconsistency may also originate from differential methods applied to the studies where one measured peptide binding affinity for the receptor ECD, while the other measured receptor activation from the full-length calcitonin and amylin receptors.

This discrepancy between ECD binding affinity and receptor activation potency may be related to the “Two-domain model” for the activation mechanism of class B G protein-coupled receptors [34,35]. The N-terminal region of peptide ligands is important for interacting with the receptor transmembrane domain for receptor activation, while the C-terminal region of the peptides is responsible for receptor ECD interaction contributing to the binding affinity of peptide ligands. Apparently, peptide interaction with receptor ECD is a part of the interaction between a full-length peptide and a full-length receptor, indicating that the limitation is clearly present in the receptor–ligand interaction studies using receptor ECDs. Consistently, there are some reports showing that the affinity changes for receptor ECDs did not represent the potency changes in full-length receptor activation [26,36].

### 4.4. Future Studies and Closing Remarks

The current study deals with amylin receptor 1 ECD interaction with peptide ligands. There are two more types of amylin receptors: amylin receptors 2 and 3. Functional amylin receptor 2/3 ECDs were reported with the ECD fusion strategy [19,20]. Peptide binding studies with amylin receptor 2/3 ECDs may provide insight into how RAMP 1/2/3 ECDs indirectly contribute to peptide ligand binding since RAMP ECD has minimal interaction with peptide ligands at amylin receptors.

Compared to the previous reports on receptor–ligand interaction, the current study addressed the mutational effects of E123 and D97 residues of the calcitonin receptor on the peptide interaction with amylin receptor 1 for the first time. In addition, this study directly measured peptide ligand affinity for the purified receptor ECDs, making a direct comparison to the wild-type receptor suitable. However, previous reports measured cAMP signaling produced from overexpressed receptors where the different expression levels of the mutated receptors could modulate cell signaling produced from the receptor. This makes the cell signaling assay suboptimal for direct comparison when receptor expression levels vary. Nevertheless, it should be noted that the results of the current study apply to the amylin receptor 1 ECD and the peptide fragments interacting with the ECD region.

Receptor–ligand interaction studies can be used as the scientific basis for the design of novel peptide ligands. For calcitonin and amylin receptors, structural information of the peptide binding mode is currently available. Biochemical and pharmacological studies that support the reported structures can help spur the development of novel peptide drugs targeting these clinically important amylin receptors.

## Figures and Tables

**Table 1:** Binding affinity of the peptide probes for amylin receptor 1 ECDs.

Figure and Peptide Probe	Amylin Receptor 1 ECD	N	pK_D_ Mean ± S.D.	Mean K_D_
FITC-sCT(22–32)	WT	3	7.09 ± 0.13	81 nM
FITC-AC413(6–25) Y25P	WT	4	7.24 ± 0.17	57 nM
Figure 3A FITC-sCT(22–32)	D101A	3	N.D.	N.D.
	D101E	3	N.D.	N.D.
	N135A	3	N.D.	N.D.
	N135D	3	N.D.	N.D.
Figure 3B FITC-AC413(6–25) Y25P	D101A	3	N.D.	N.D.
	D101E	3	N.D.	N.D.
	N135A	3	N.D.	N.D.
	N135D	3	N.D.	N.D.
Figure 4A FITC-sCT(22–32)	E123A	3	6.44 ± 0.27 ^a^	365 nM
	E123D	3	7.28 ± 0.21 ^c^	53 nM
	N124A	3	N.D.	N.D.
	N124D	3	7.29 ± 0.19	52 nM
Figure 4B FITC-AC413(6–25) Y25P	E123A	3	6.89 ± 0.17 ^b^	129 nM
	E123D	3	6.87 ± 0.20 ^b^	136 nM
	N124A	3	6.33 ± 0.08 ^b^	467 nM
	N124D	3	6.09 ± 0.10 ^b^	822 nM
Figure 5A FITC-sCT(22–32)	D97A	3	6.26 ± 0.23 ^a^	548 nM
	D97E	3	6.79 ± 0.16 ^d^	161 nM
Figure 5B FITC-AC413(6–25) Y25P	D97A	3	7.21 ± 0.10	62 nM
	D97E	3	7.09 ± 0.12	81 nM

N.D., not determined due to lacking significant binding with the tested receptor concentrations. When the mutated receptor ECDs failed to achieve 50% of the anisotropy increase in the wild-type receptor ECD (about less than 0.1 anisotropy value obtained at the maximal concentrations used), the pK_D_ values were not determined. ^a^ *p* < 0.05 by one-way ANOVA with Dunnett’s post hoc test. FITC-sCT(22–32) affinity for amylin receptor 1 ECD wild-type (WT) was used as a control group for Dunnett’s post hoc test. ^b^ *p* < 0.05 by one-way ANOVA with Dunnett’s post hoc test. FITC-AC413(6–25) Y25P affinity for amylin receptor 1 ECD WT was used as a control group for Dunnett’s post hoc test. From the binding curves, apparent affinity differences between the mutations introduced to one receptor residue were further analyzed with statistical analysis with Tukey’s post hoc test. ^c^ *p* < 0.05 by one-way ANOVA with Tukey’s post hoc test compared to the FITC-sCT(22–32) E123A group. ^d^ Not significant by one-way ANOVA with Tukey’s post hoc test compared to the FITC-sCT(22–32) D97A group.

## Data Availability

Raw data will be made available upon reasonable requests to the corresponding author.

## Supplementary Materials

The following supporting information can be downloaded at: https://www.mdpi.com/article/10.3390/biomedicines13071787/s1, Figure S1: SEC elution profiles of wild-type (WT) and mutated amylin receptor 1 ECDs used for the current study; Figure S2: SDS-PAGE of purified/glycosylated amylin receptor 1 ECDs used for this study; Figure S3: Competition binding assay with a non-labeled salmon calcitonin (sCT) (22–32) fragment for the amylin receptor 1 ECD with the E123A or E123D mutation; Table S1: Expression and purification profiles of purified amylin receptor 1 ECDs; Table S2: Fluorescence intensity changes and maximum receptor concentrations used for peptide ligand binding assay.
