# Supplementary material for: Amylin Receptor 1 Mutagenesis Revealed a Potential Role of Calcitonin Serine 29 in Receptor Interaction
Source: Biomedicines. 2025 Jul 21;13(7):1787. doi: 10.3390/biomedicines13071787 (PMC12292835; doi:10.3390/biomedicines13071787)
Supplement: Supplementary file 1 [file biomedicines-13-01787-s001.zip › biomedicines-3731969-supplementary.pdf]

Supplemental document

Title: Amylin Receptor 1 Mutagenesis Revealed a Potential Role of Calcitonin Serine 29 in Receptor Interaction

Author: Hyeseon Song, Jaehyeok Jang, Minjae Park, Junsu Yun, Jeongwoo Jin and Sangmin Lee*

Department of Medicinal Biotechnology, College of Health Science, Dong-A University, Busan, Republic of Korea, 49315

* Correspondence: smlee1981@dau.ac.kr; Address: S09-0313, 37, Nakdong-daero 550beon-gil, Saha-gu, Busan, Republic of Korea, 49315; Tel.: 82-51-200-7566

Table S1. Expression and purification profiles of purified amylin receptor 1 ECDs

Purified amylin receptor 1 ECD	Peak elution volume from SEC profile (mL)	Total yield (mg)	% of WT yield	Experiment number
Wild-type (WT)	82.65	0.718	100	H-SL557
D101A	81.22	0.12	16.7	D-JJ052
D101E	81.00	0.205	28.6	D-JJ032
N135A	81.36	0.141	19.6	D-SL033
N135D	80.81	0.095	13.2	D-SL022
E123A	81.57	0.169	23.5	D-JJ059
E123D	81.71	0.224	31.2	D-SL021
N124A	81.96	0.043	6.0	D-JS046
N124D	82.17	0.107	14.9	D-JS049
D97A	81.55	0.04	5.6	D-SL089
D97E	80.45	0.142	19.8	D-JS035

ECD, extracellular domain. SEC, size exclusion chromatography.

Table S2. Fluorescence intensity changes and maximum receptor concentrations used for peptide ligand binding assay

Purified amylin receptor 1 ECD	Average fluorescence intensity change of FITC-sCT(22–32) upon receptor ECD binding (% of a free probe intensity, Mean ± S.D.)	The maximum concentration used for the binding assay (μM)
WT	71 ± 4%	3
D101A	101 ± 4%	1
D101E	118 ± 13%	3
N135A	81 ± 10%	3
N135D	99 ± 0.1%	3
E123A	72 ± 1%	3
E123D	72 ± 6%	3
N124A	85 ± 11%	1
N124D	77 ± 5%	3
D97A	75 ± 12%	3
D97E	78 ± 4%	3

Purified amylin receptor 1 ECD	Average fluorescence intensity change of FITC-AC413(6–25) Y25P upon receptor ECD binding (% of a free probe intensity, Mean ± S.D.)	The maximum concentration used for the binding assay (μM)
WT	67 ± 8%	1
D101A	97 ± 3%	1
D101E	99 ± 3%	1
N135A	87 ± 15%	1
N135D	85 ± 15%	1
E123A	77 ± 13%	1
E123D	72 ± 4%	1
N124A	87 ± 2%	1
N124D	88 ± 8%	1
D97A	71 ± 4%	1
D97E	68 ± 3%	1

ECD, extracellular domain. FITC, fluorescein isothiocyanate. sCT, salmon calcitonin. S.D., standard deviation.

Figure S1. SEC elution profiles of wild-type (WT) and mutated amylin receptor 1 ECDs used for the current study

A) WT

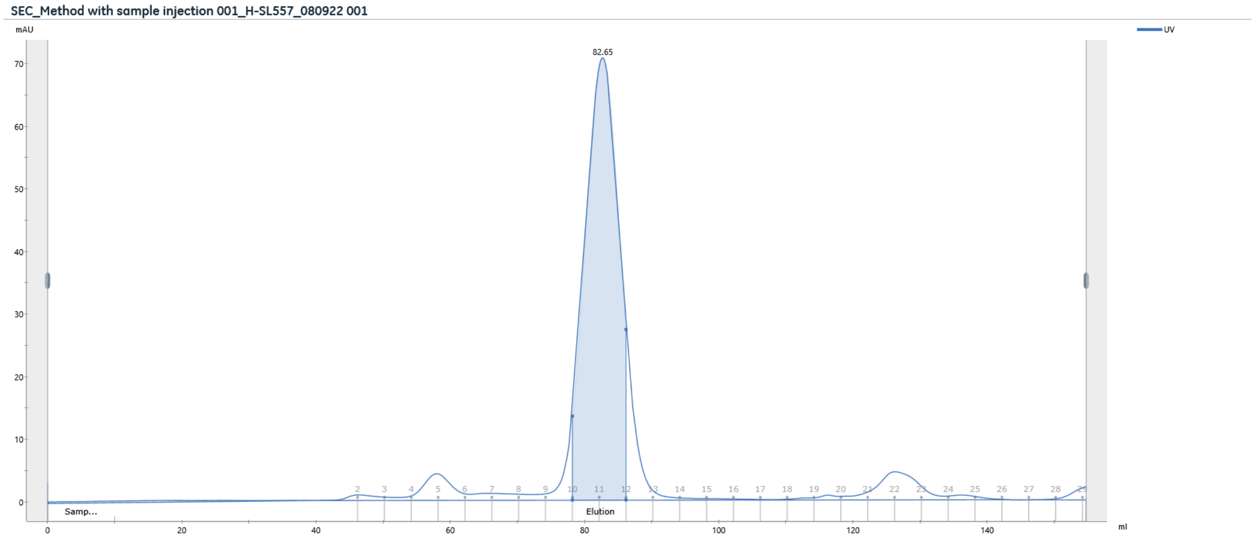

B) D101A

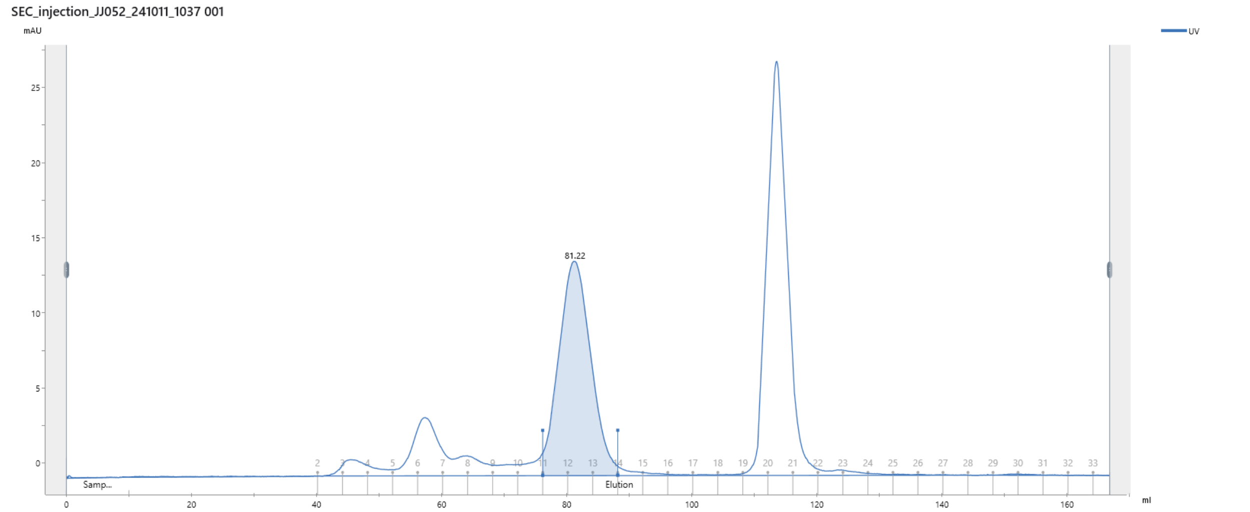

Peak Table - UV

Peak	Retention ml	Area ml*mAU	Area %	Ext coeff. mg^{-1} ml cm^{-1}	Fraction(s)	Volume ml	Conductivity mS/cm
Peak A	81.221	84.02	100		11 - 13	12.001	12.73

C) D101E

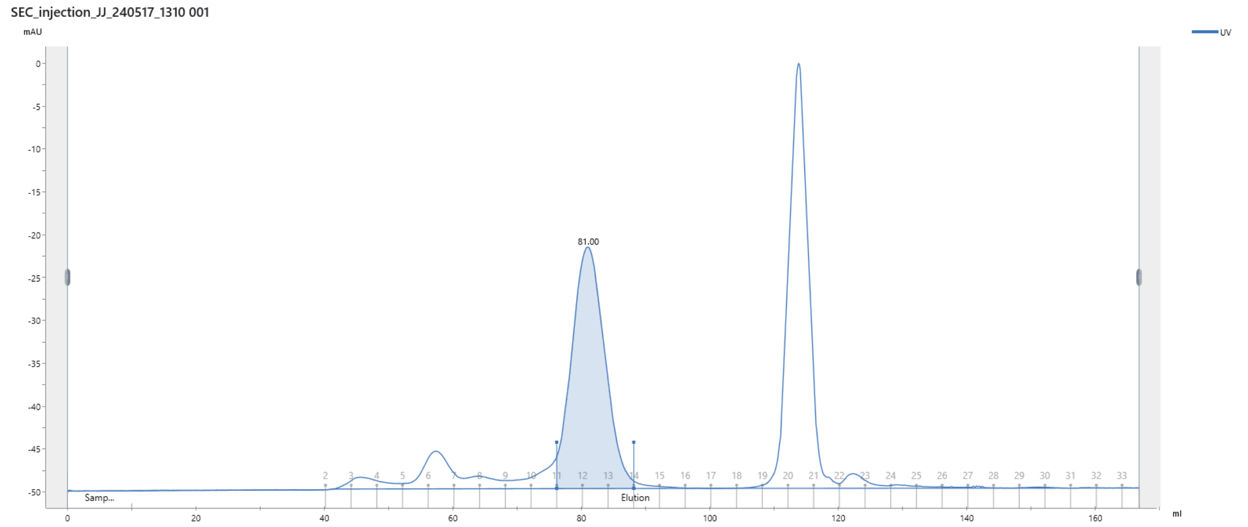

Peak Table - UV

Peak	Retention ml	Area ml*mAU	Area %	Ext coeff. mg $^{-1}$ ml cm $^{-1}$	Fraction(s)	Volume ml	Conductivity mS/cm
Peak A	80.997	167.1	100		11 - 13	12.001	12.33

D) N135A

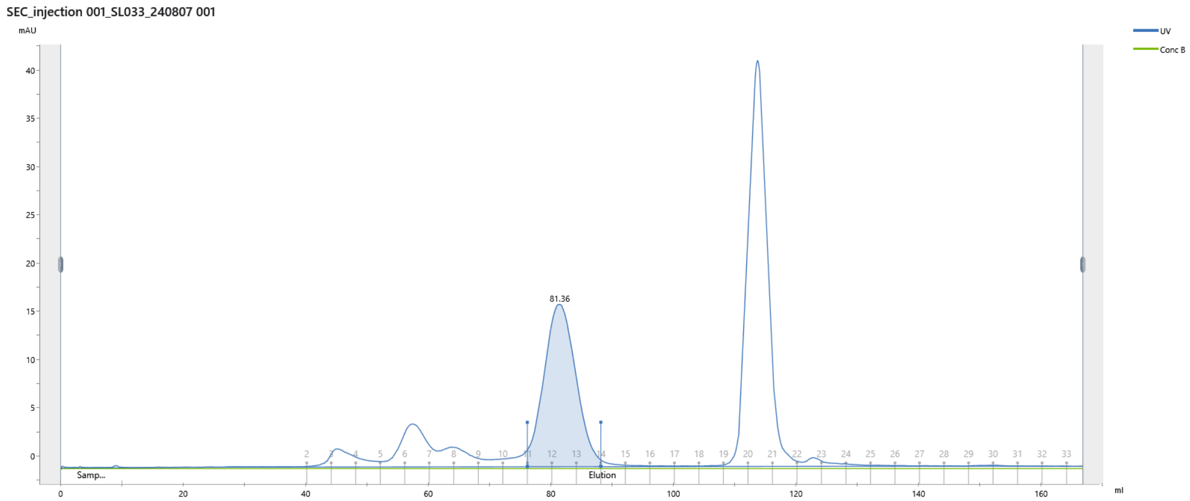

E) N135D

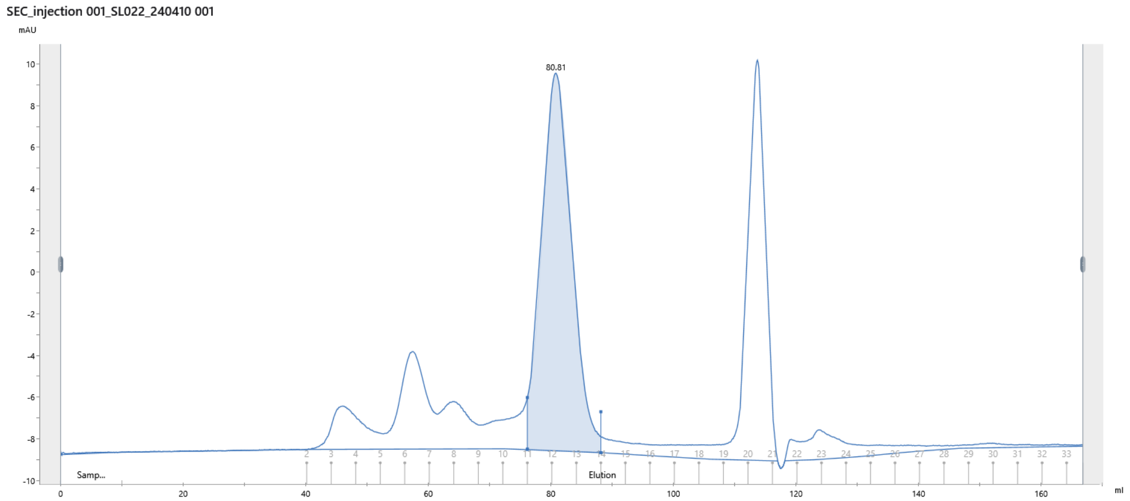

F) E123A

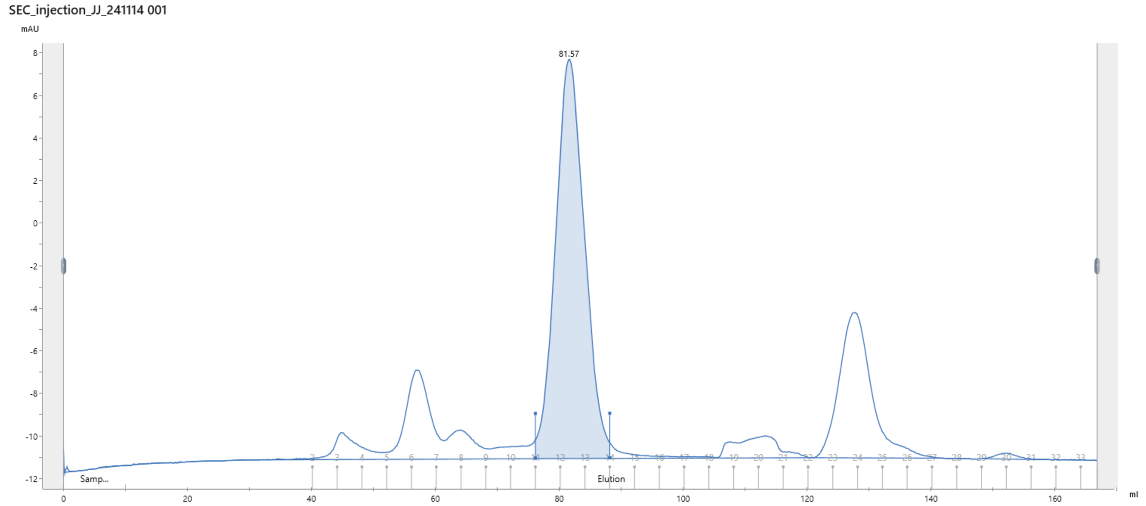

Peak Table - UV

Peak	Retention ml	Area ml*mAU	Area %	Ext coeff. mg^{-1} ml cm^{-1}	Fraction(s)	Volume ml	Conductivity mS/cm
Peak A	81.571	101.7	100		11 - 13	12.002	12.85

G) E123D

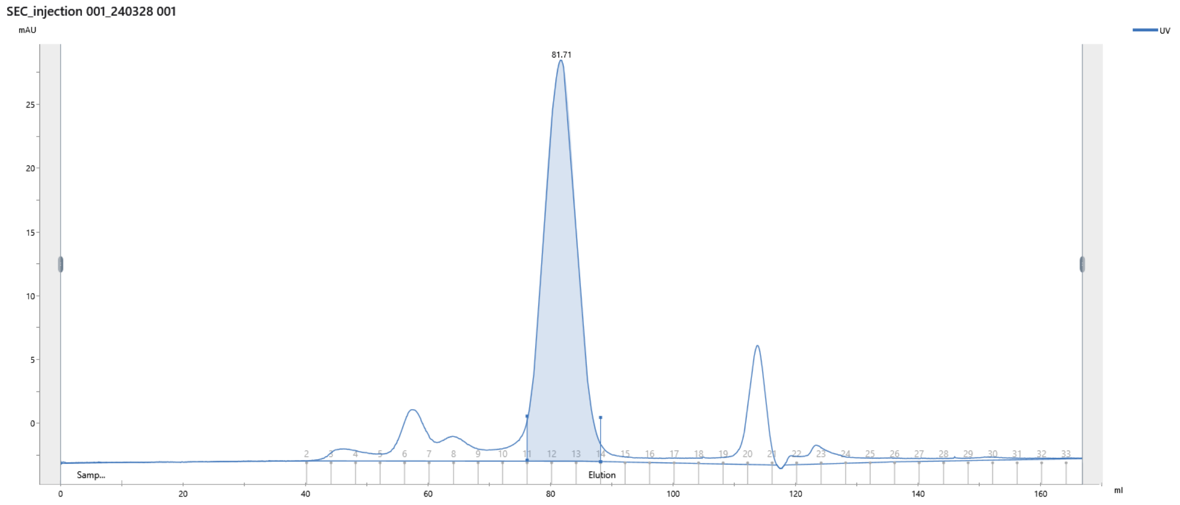

H) N124A

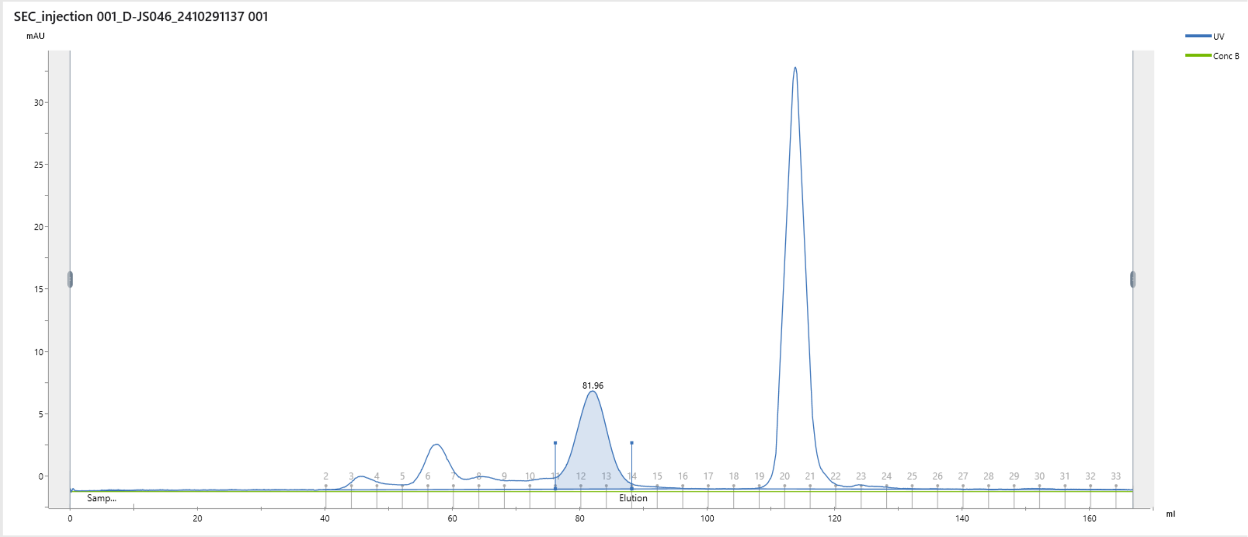

I) N124D

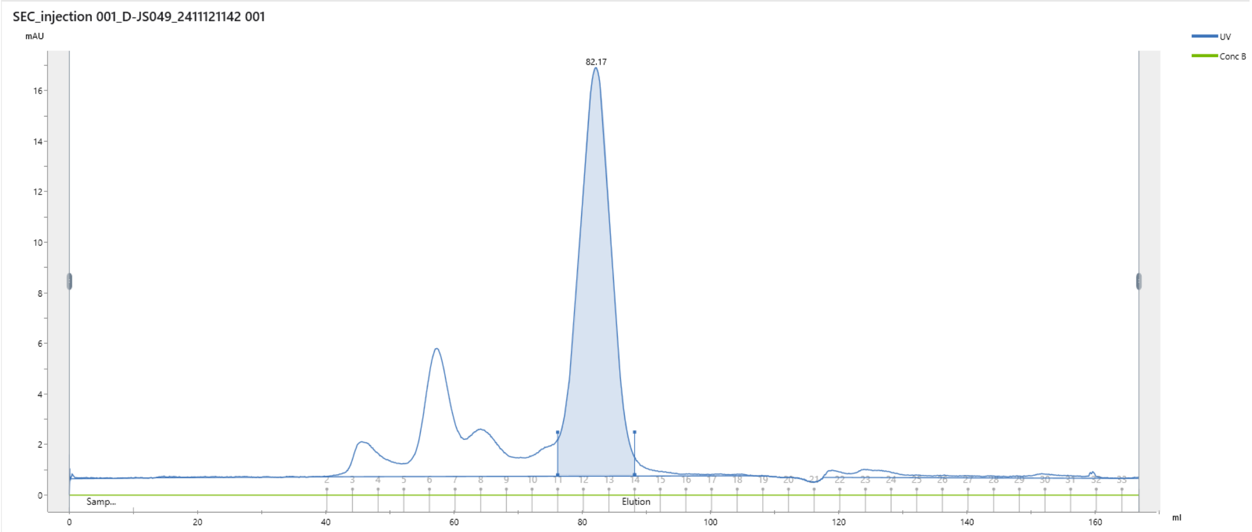

J) D97A

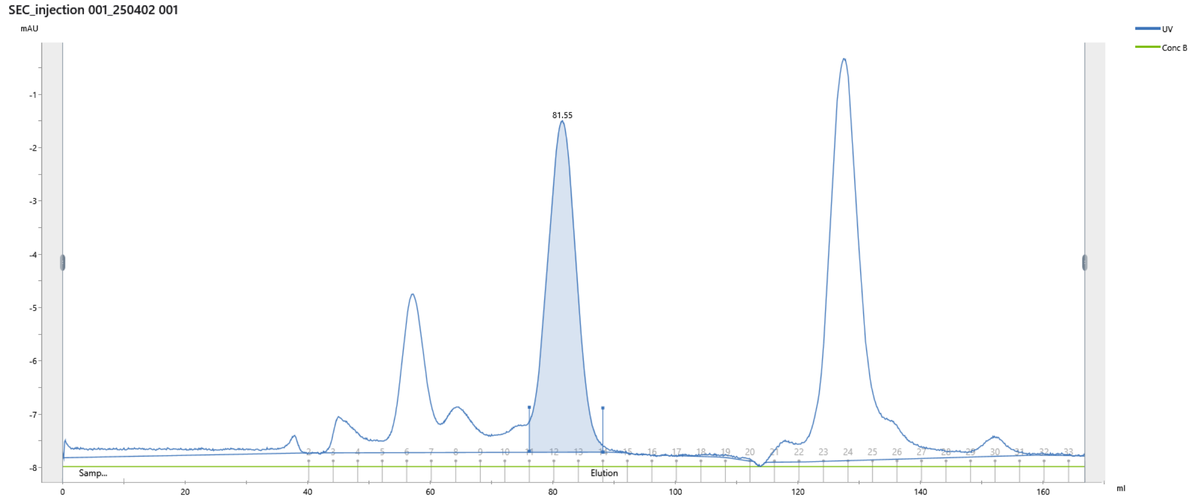

K) D97E

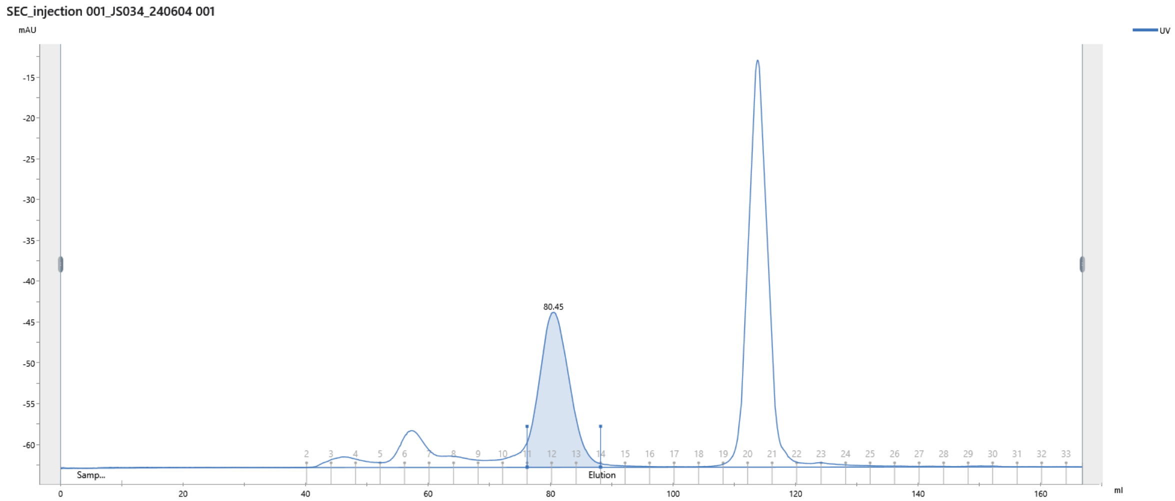

The fractions corresponding to the blue area were collected as the final purified amylin receptor 1 ECD proteins for storage. The peak elution volume of the elution profile was shown on the top of the peak colored in blue. ECD, extracellular domain. SEC, size exclusion chromatography.

Figure S2. SDS-PAGE of purified/glycosylated amylin receptor 1 ECDs used for this study

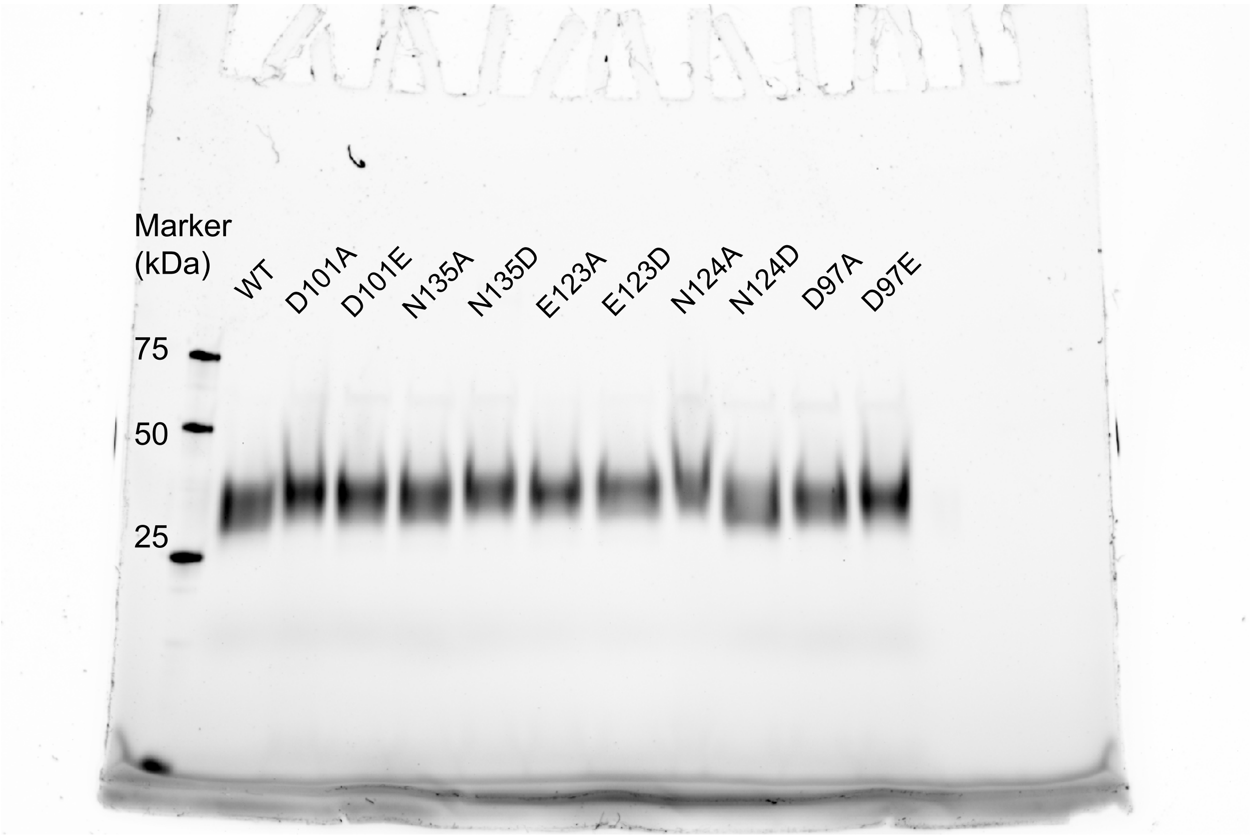

Total 4 μg of wild-type and mutated purified amylin receptor 1 ECDs were loaded to a Mini-PROTEAN TGX 4–15 % Stain-Free gel (Bio-Rad, Cat.#4568086, 15 wells/gel). The SDS-PAGE clearly shows that the amylin receptor 1 ECDs used in the current study have high purity and a similar molecular weight. The gel was run by 180 mV for 30 min with the 1X Tris-Glycine running buffer (25 mM Tris, 192 mM glycine, and 0.1 % (w/v) SDS) diluted from the 10X Tris-Glycine running buffer and with a Mini-PROTEAN Tetra Cell, 4-Gel system (Bio-Rad, Cat.# 1658004). After running, the image was obtained by using a GelDoc Go gel imaging system (Bio-Rad) (with a protein gel option for stain-free gels, 5 min UV light activation). The mutation introduced to the calcitonin receptor ECD was shown on the top of the bands in the gel. Precision Plus Protein™ WesternC™ Blotting Standards (Bio-Rad, Cat.#1610376) were used to show protein markers. WT, wild-type. ECD, extracellular domain.

Figure S3. Competition binding assay with a non-labeled salmon calcitonin (sCT) (22–32) fragment for the amylin receptor 1 ECD with the E123A or E123D mutation.

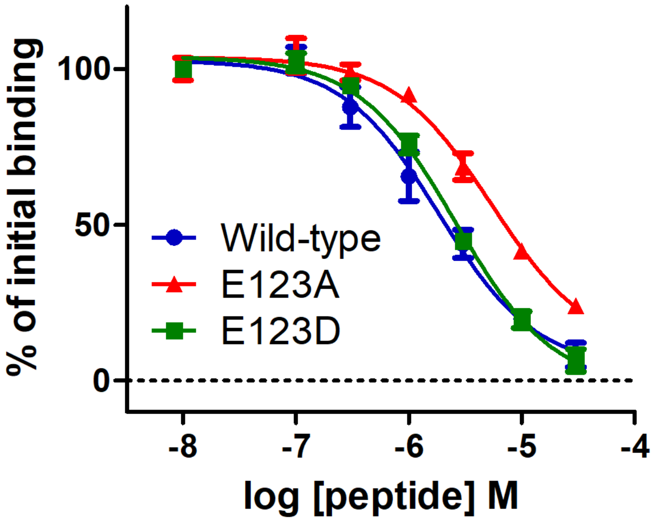

pK_I values of sCT(22–32) for the wild-type amylin receptor 1 ECD, the ECD with the E123A mutation, and the ECD with the E123D mutation were 6.05 (K_I 891 nM), 5.60 (K_I 2.5 μM), and 5.91 (K_I 1.2 μM), respectively. The mutational pattern was conserved in the competition binding assay with a non-labeled peptide probe when it was compared to the results shown in Figure 4A. These results suggest that FITC labeling to the peptide probes would not intervene the receptor mutational effects on the peptide probe binding affinity. Competition binding assay was performed at the receptor ECD concentration corresponding to K_D values obtained from the saturation binding (Wild-type 81 nM, E123A 365 nM, and E123D 53 nM). FITC-sCT(22–32) 10 nM was used as a peptide probe for the competition binding assay. The mixture of the competitive peptide with varying concentrations, 10 nM FITC-sCT(22–32), and the receptor ECD protein with the concentration described earlier was incubated for 1 hour. The anisotropy signal was measured by using a BioTek Synergy H1 microplate reader (Agilent, Santa Clara, CA, USA). The initial anisotropy with the lowest sCT(22–32) concentration used for the competition binding assay was assumed as 100 %. The anisotropy value produced by the free FITC-sCT(22–32) probe in the absence of the receptor ECD was used as 0 %. sCT(22–32) was custom-synthesized from Genscript

(Piscataway, NJ, USA). The HPLC purity of sCT(22–32) was 99.9% and its molecular mass was validated by Genscript with mass spectrometry. The concentration of sCT(22–32) was measured with the absorbance at 280 nm and its extinction coefficient (1490 $M^{-1} \cdot cm^{-1}$). ECD, extracellular domain.
